# Supplementary material for: 3D bioprinted multilayered cerebrovascular conduits to study cancer extravasation mechanism related with vascular geometry
Source: Nat Commun. 2023 Nov 24;14:7696. doi: 10.1038/s41467-023-43586-4 (PMC10673893; doi:10.1038/s41467-023-43586-4)
Supplement: Supplementary file 1 — Supplementary Information [file 41467_2023_43586_MOESM1_ESM.pdf]

Supplementary Information

**Supplementary Figure 1**	Biochemical components in brain-derived decellularized extracellular matrix (BdECM).
**Supplementary Figure 2**	Protein composition of brain-derived decellularized extracellular matrix (BdECM).
**Supplementary Figure 3**	Rheological property of brain-derived decellularized extracellular matrix (BdECM) pre-gel.
**Supplementary Figure 4**	Viability and proliferation rate of human-brain microvascular endothelial cells (HBMECs) in brain-derived decellularized extracellular matrix (BdECM)-based bioinks.
**Supplementary Figure 5**	Effects of alginate in hybrid brain-derived decellularized extracellular matrix (BdECM) bioink on cellular morphology.
**Supplementary Figure 6**	Comparison of mechanical and rheological properties of native brain tissue and hybrid brain-derived decellularized extracellular matrix (BdECM) bioink.
**Supplementary Figure 7**	Density (weight/volume) of native porcine brain.
**Supplementary Figure 8**	The experimental timeline for metastatic cancer progression study.
**Supplementary Figure 9**	Structural maintenance of mature multilayered cerebrovascular conduit depending on the presence of the housing and the bath environment.
**Supplementary Figure 10**	Tissue maturation in the *in vitro* brain metastasis model over 14 days.
**Supplementary Figure 11**	Quantitative reverse-transcription polymerase chain reaction results demonstrating the cerebrovascular tissue formation.
**Supplementary Figure 12**	The relative average fluorescence intensity of the diffused permeability probes in the 3D bioprinted cerebrovascular conduits with various geometries.
**Supplementary Figure 13**	Trans-epithelial electrical resistance (TEER) analysis of multilayered cerebrovascular conduits (MCCs).
**Supplementary Figure 14**	Tumor cell adhesion in multilayered cerebrovascular conduits with different angles.

Supplementary Figure 15 Tumor cell adhesion on the confluent cerebrovascular wall.

Supplementary Figure 16 Extravasation of introduced tumor cells in multilayered cerebrovascular conduits over time.

Supplementary Figure 17 Tumor cell extravasation from a multilayered cerebrovascular conduit (MCC).

Supplementary Figure 18 Blocking cell adhesion molecules and cancer cell adhesion.

Supplementary Figure 19 Expression of adhesion proteins in multilayered cerebrovascular conduits in response to the different types of cancer cells.

Supplementary Figure 20 Fluid dynamics simulation of pressure in the metastatic cancer model.

Supplementary Figure 21 Generation of the pressure gradient at different fluid flow velocities.

Supplementary Figure 22 Quantitative reverse-transcription PCR results demonstrating the effects of coculture and vascular curvatures on metastatic brain cancer development.

Supplementary Figure 23 The bioreactor set-up for development of an *in vitro* metastatic cancer model.

Supplementary Table 1 Protein expression level of cell adhesion molecules.

Supplementary Table 2 Cellular information of circulating tumor cells.

Supplementary Table 3 Quantitative reverse-transcription PCR primer sequences.

Supplementary Figure 1. Biochemical components in brain-derived decellularized extracellular matrix (BdECM). a, b) Major ECM components including glycosaminoglycans (GAGs) and collagen are maintained, whereas c) dsDNA is eliminated after decellularizing the native brain tissue. The results show mean ± SD from n = 3 samples. The significance is determined using paired t-test (***, p ≤ 0.001; **, p ≤ 0.01). Source data are provided as a Source Data file.

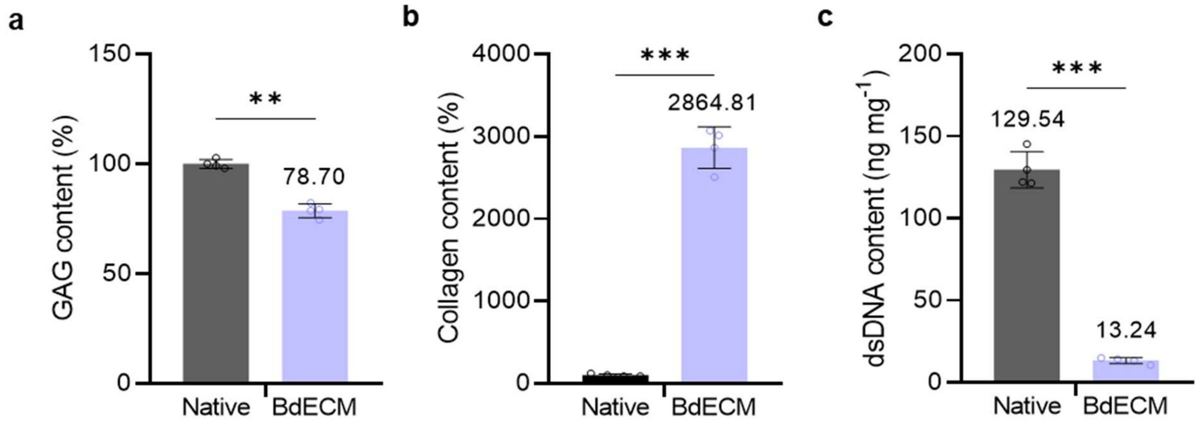

Supplementary Figure 2. Protein composition of brain-derived decellularized extracellular matrix (BdECM). a) The proteomic analysis of BdECM reveals the presence of over 2,000 proteins. b) The top 10 proteins are COL1A1, HSPG2, PLP1, COL4A2, COL1A2, LAMB2, SYN1, FN1, FBN1, and FGA. c) Basement membrane components such as HSPG2, COL4A2, laminins (LAMB2 and LAMC1) and nidogen are detected. d) Interstitial matrix proteins including proteoglycans, tenascins, fibrous collagens, and adhesive glycoproteins are identified. e) More than 20 different collagen types, including collagen type I alpha I (COL1A1), collagen type IV alpha II (COL4A2), and collagen type I alpha II (COL1A2), are discovered and the components constitute over 30% of the total composition of BdECM. Source data are provided as a Source Data file.

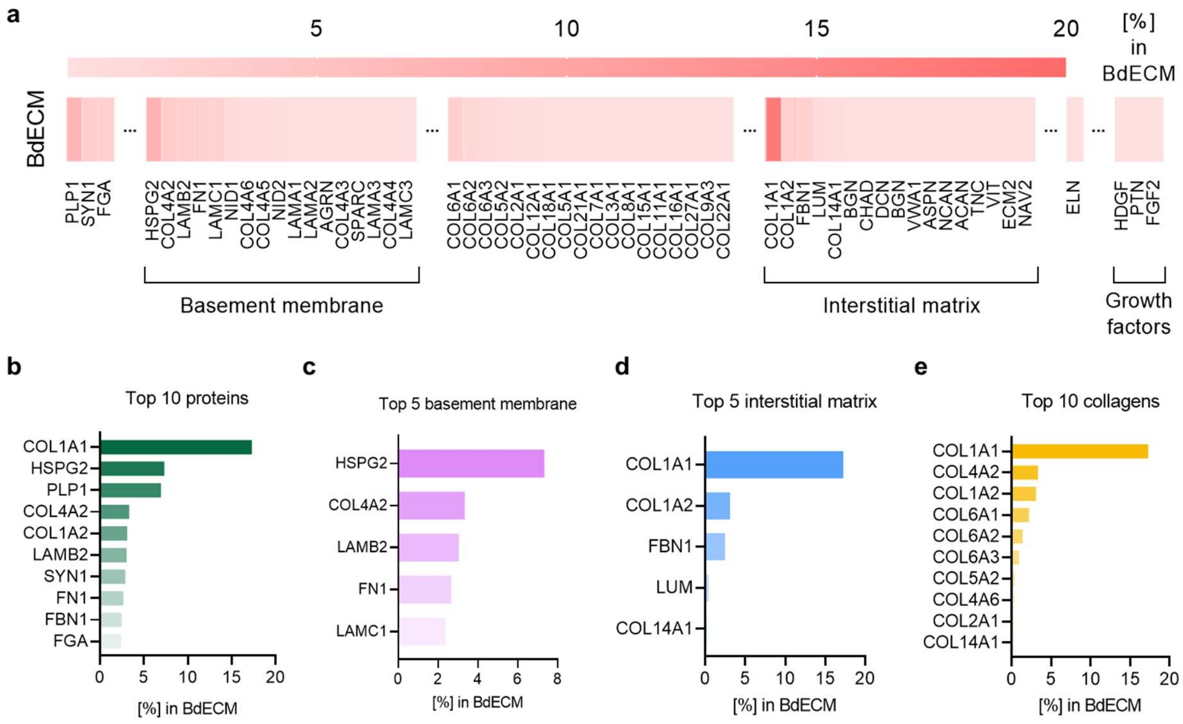

Supplementary Figure 3. Rheological property of brain-derived decellularized extracellular matrix (BdECM) pre-gel. a) The stable sol-gel transition behavior of BdECM pre-gel. G' and G'' represent storage modulus and loss modulus, respectively. The results are compiled from n = 3 samples. Lines connect mean values. b) Thermal gelation kinetics of the BdECM pre-gel. Source data are provided as a Source Data file.

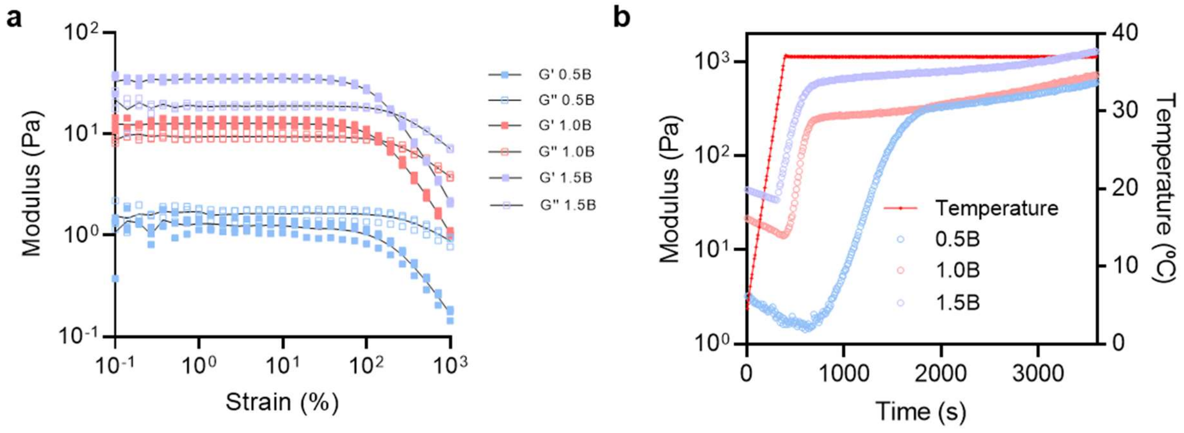

Supplementary Figure 4. Viability and proliferation rate of human-brain microvascular endothelial cells (HBMECs) in brain-derived decellularized extracellular matrix (BdECM)-based bioinks. The optimal formulation of hybrid BdECM bioink is verified through a) live/dead assay and b) CCK-8 assay. BdECM and alginate are labeled as B and A, respectively. The results show mean ± SD from *n* = 3 samples. The significance is determined using ordinary one-way ANOVA (***, $p \leq 0.001$). Source data are provided as a Source Data file.

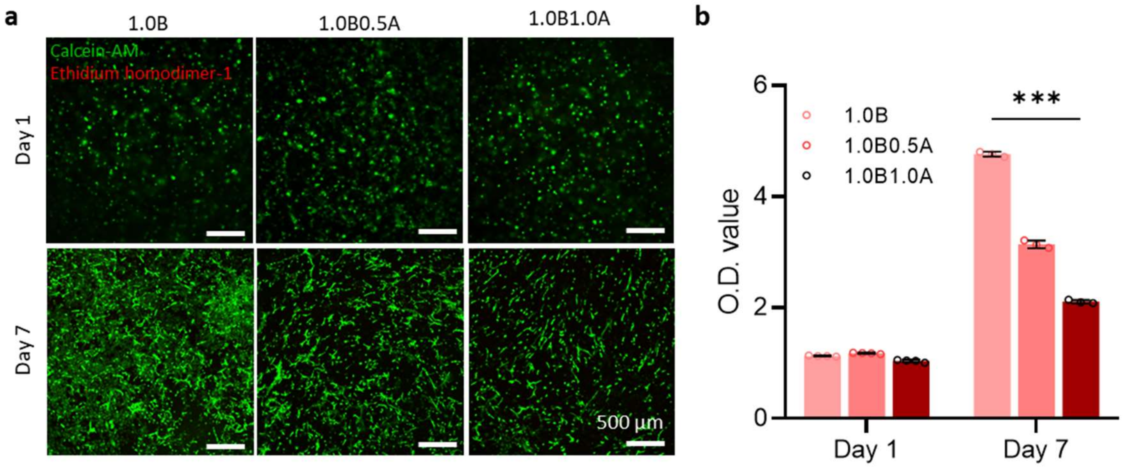

Supplementary Figure 5. Effects of alginate in hybrid brain-derived decellularized extracellular matrix (BdECM) bioink on cellular morphology. Although a) human brain microvascular endothelial cells and b) neural progenitor cells in hybrid BdECM bioink (1.0B0.5A) have relatively thinner and longer shapes and more pseudopodia than those in pure BdECM bioink (1.0B) (day 7), the morphological gap has decreased as the cells proliferate (day 14). BdECM and alginate are labeled as B and A, respectively.

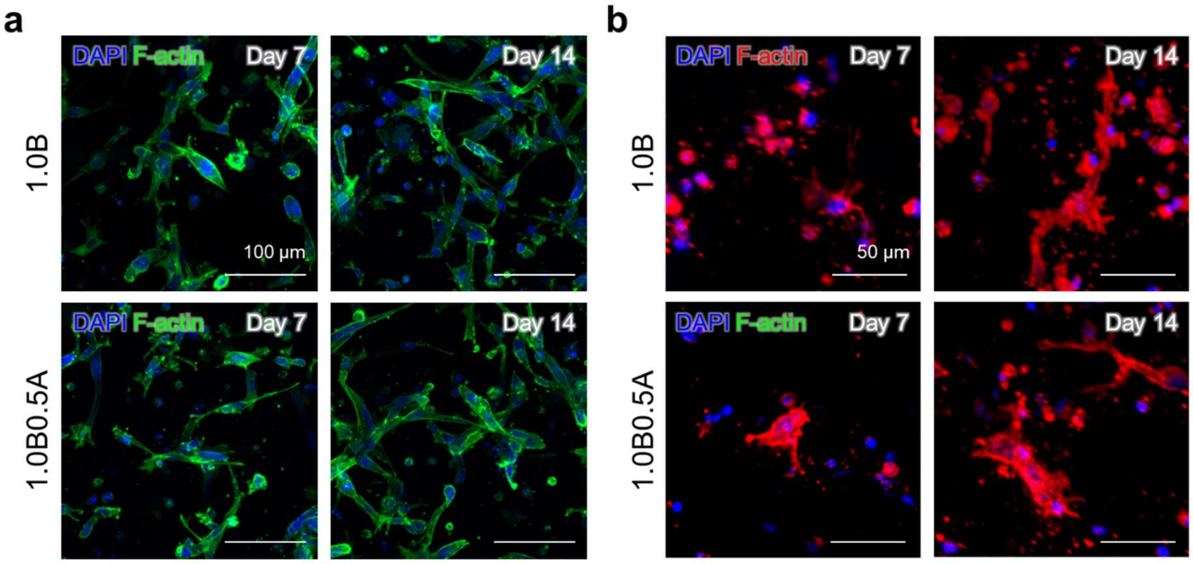

Supplementary Figure 6. Comparison of mechanical and rheological properties of native brain tissue and hybrid brain-derived decellularized extracellular matrix (BdECM) bioink. a) Mechanical and rheological properties of the native brain tissue and the multilayered cerebrovascular conduits (MCCs) are measured. b) The compressive modulus of the native tissue and MCCs were 118.28 ± 6.16 Pa and 85.26 ±11.97 Pa, respectively. The results show mean ± SD from n = 5 samples. The significance is determined using two-tailed t-test (***, $p \leq 0.001$). c) The storage modulus (G') and loss modulus (G'') of the native tissue were 3104.62 ± 2018.61 Pa and 729.72 ± 644.29 Pa, respectively, while those of MCCs are 1377.51 ± 1796.84 Pa and 266.52 ± 435.58 Pa, respectively. The results are compiled from n = 3 samples. Source data are provided as a Source Data file.

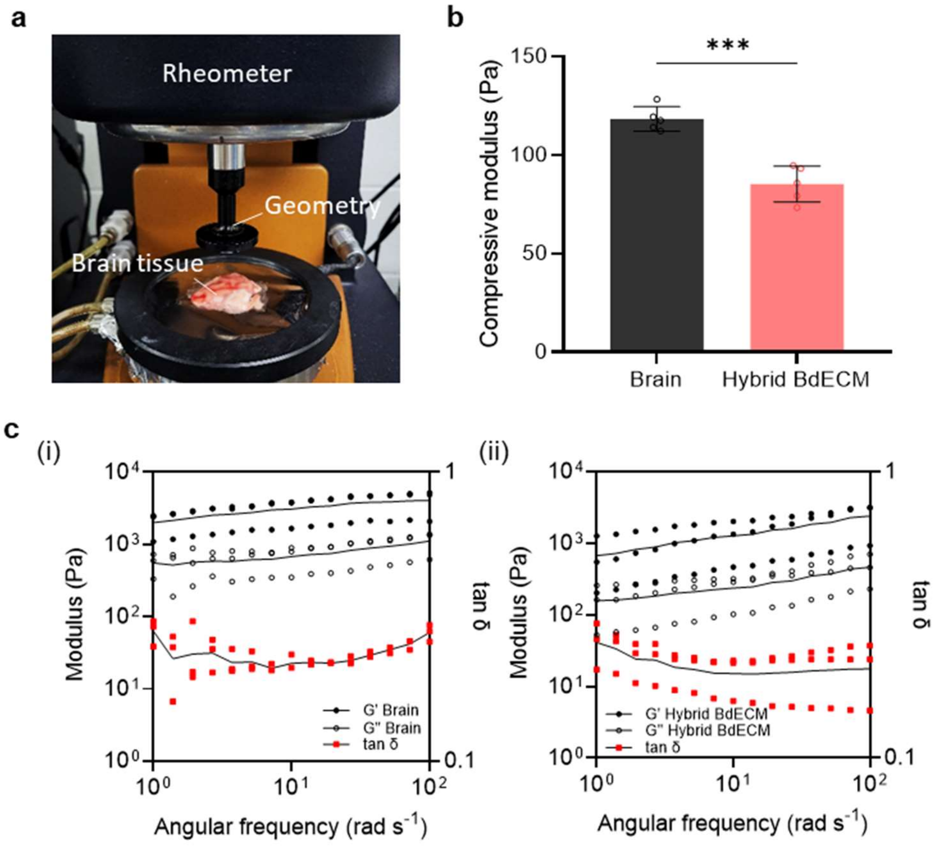

Supplementary Figure 7. Density (weight/volume) of native porcine brain. The results show mean ± SD from n = 3 samples. Source data are provided as a Source Data file.

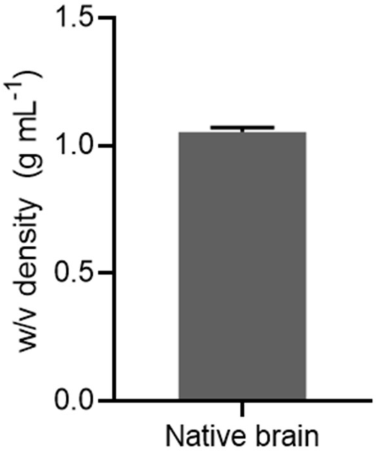

Supplementary Figure 8. The experimental timeline for metastatic cancer progression study. After in-bath 3D triaxial bioprinting of multilayered cerebrovascular conduits (MCCs) using the brain endothelial cells (BECs), brain pericytes (BPCs), and neural progenitor cells (NPCs), the constructs are stabilized and cultured. Subsequently, after the MCCs mature for 14 days, circulating tumor cells are perfused into MCCs to investigate the hemodynamic effects on metastatic cancer progression. On day 1, 7, 14, and 15, the samples are harvested and the functionality of the model for mechanism study is verified.

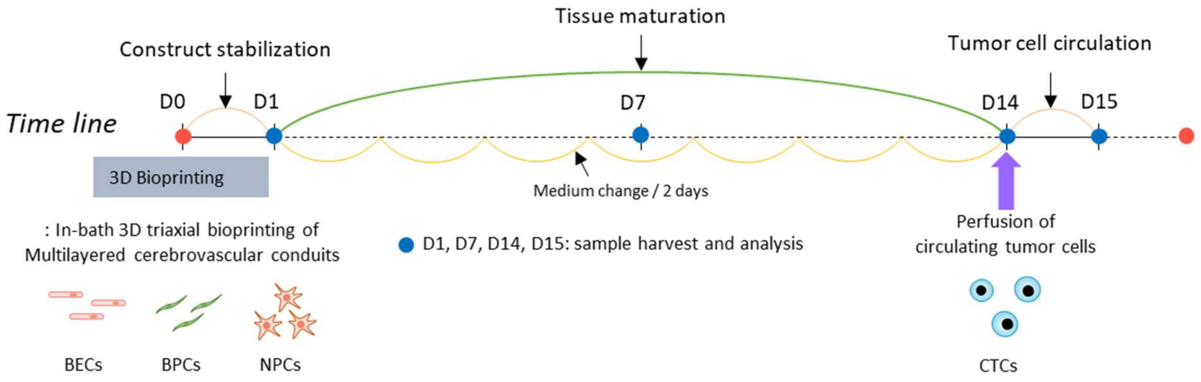

Supplementary Figure 9. Structural maintenance of mature multilayered cerebrovascular conduit depending on the presence of the housing and the bath environment.

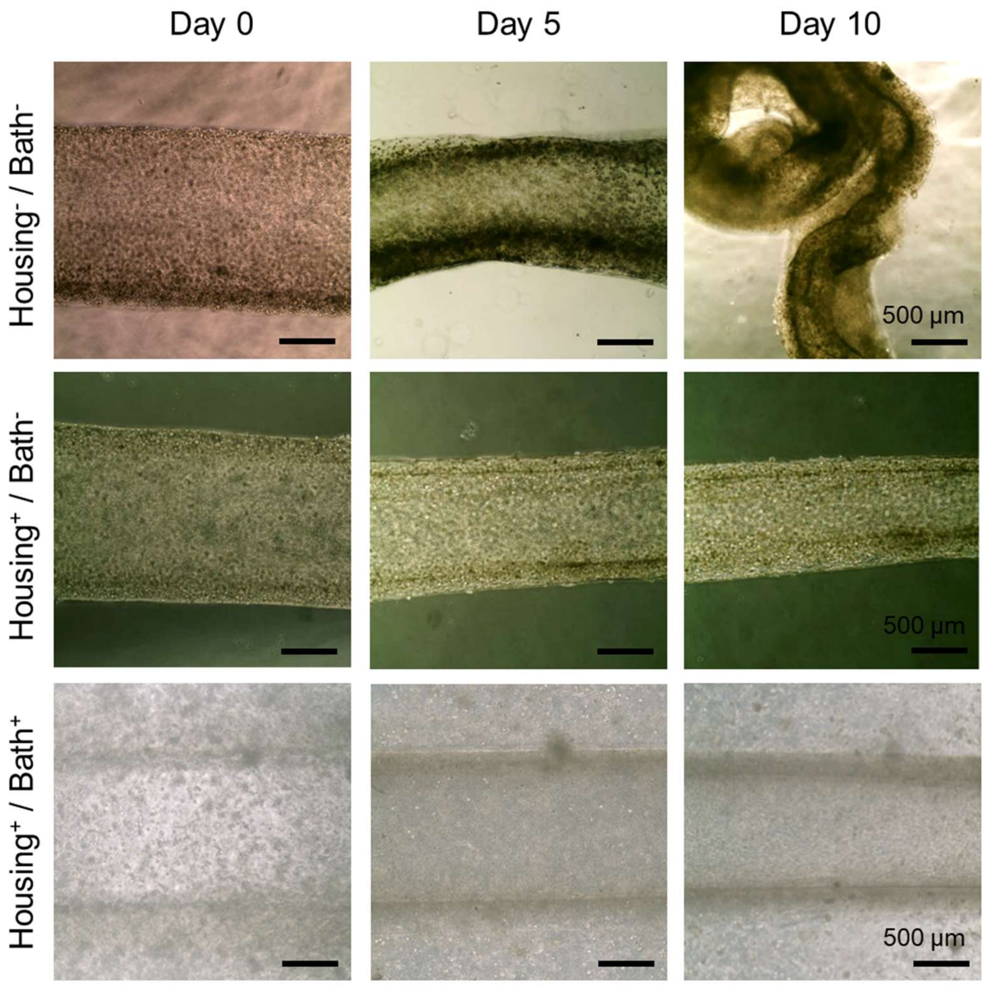

Supplementary Figure 10. Tissue maturation in the *in vitro* brain metastasis model over 14 days. a) The triple layers of the cerebrovascular conduits have been maintained for 14 days. The scale bar is 500 µm. b) The encapsulated cells have stretched their bodies, forming the mature endothelium, the confluent pericytes, and the differentiated neural progenitor cells into the astrocytes and the neurons. The brain endothelial cells, the brain pericytes, the neural progenitor cells, the astrocytes, and the nerve cells are indicated in the figure as BEC, BPC, NPC, AC, and NC, respectively. The scale bars are 50 µm.

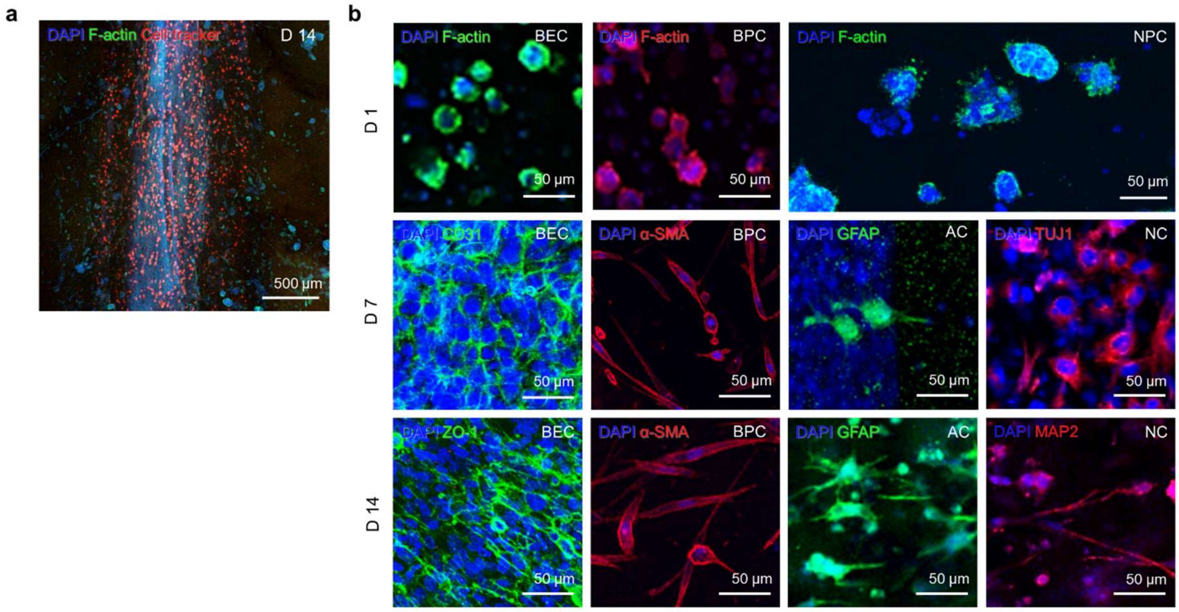

Supplementary Figure 11. Quantitative reverse-transcription polymerase chain reaction results demonstrating the cerebrovascular tissue formation. The gene expression of maturation markers (endothelial tight junction markers, *ZO-1* and *Occludin*; pericyte markers, *α-SMA* and *PDGFR-β* an astrocytic marker, *GFAP*; neuronal markers, *TUJ1* and *MAP2*) increases over time. The results show mean ± SD from $n = 3$ samples. The significance is determined using two-tailed t-test (**, $p \leq 0.01$; *, $p \leq 0.05$). Source data are provided as a Source Data file.

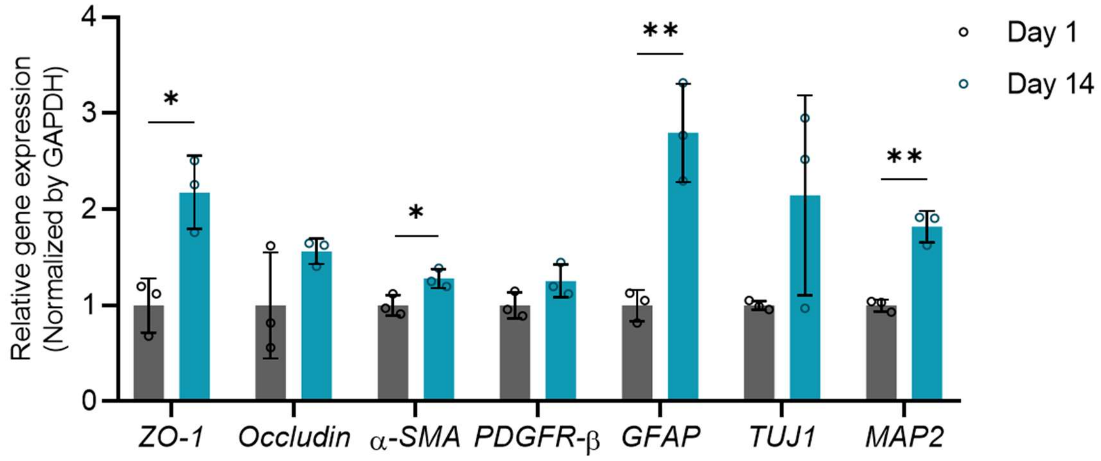

Supplementary Figure 12. The relative average fluorescence intensity of the diffused permeability probes in the 3D bioprinted cerebrovascular conduits with various geometries. a) After the fluorescein isothiocyanate (FITC)-dextran particles are perfused, and the diffusion level is imaged, over time. b) The relative fluorescence intensity increases over time, but there is no significant difference among the groups (0°, 90°, and 150°). The results show mean ± SD from n = 3 samples. The significance is determined using ordinary one-way ANOVA (**, p ≤ 0.01; ns, no significance). Source data are provided as a Source Data file.

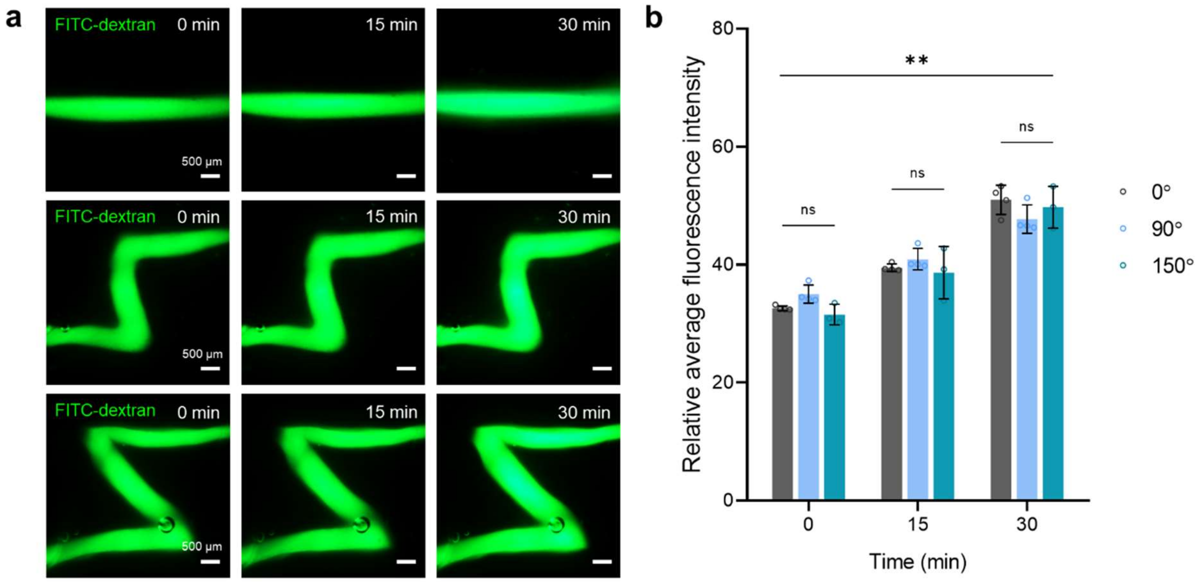

Supplementary Figure 13. Trans-epithelial electrical resistance (TEER) analysis of multilayered cerebrovascular conduits (MCCs). a) To verify the effects of coculture condition, electrodes are placed on either side of the mature MCCs constituted of brain endothelial cells (BECs), brain pericytes (BPCs), and neural progenitor cells (NPCs). b) The lowest TEER value is observed in the monoculture group containing only BECs (BEC^+ BPC^- NPC^-). When co-cultured with BPCs (BEC^+ BPC^+ NPC^-), the TEER value increases. Furthermore, the TEER value increases even more when the differentiated NPCs are also included in the co-culture (BEC^+ BPC^+ NPC^+). The results show mean ± SD from $n = 11$ (BEC^+ BPC^- NPC^- and BEC^+ BPC^+ NPC^-) and $n = 7$ (BEC^+ BPC^+ NPC^+) samples. The significance is determined using two tailed t-test (***, $p \leq 0.001$). Source data are provided as a Source Data file.

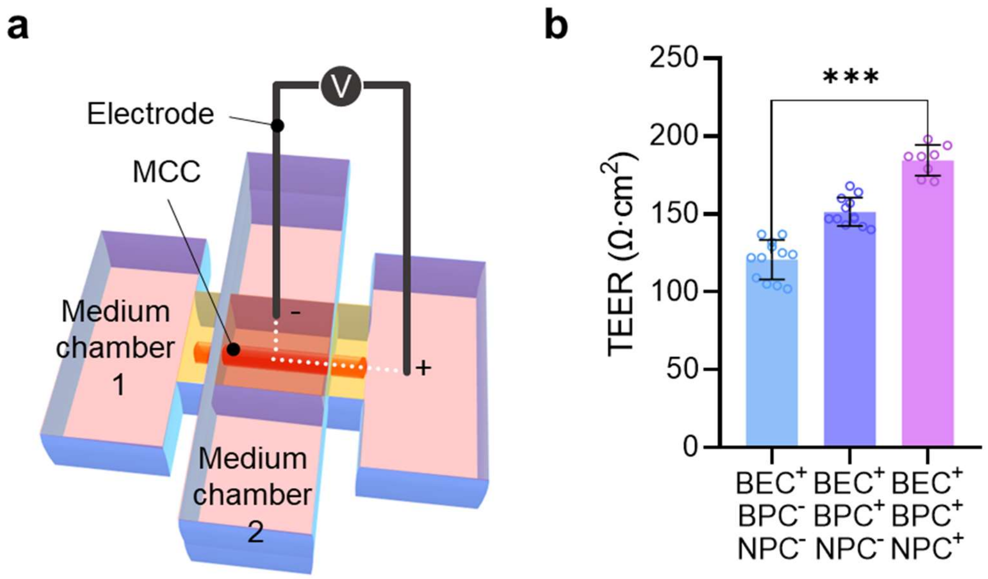

Supplementary Figure 14. Tumor cell adhesion in multilayered cerebrovascular conduits with different angles. On the confluent endothelium, cancer cells adhere to initiate the metastasis.

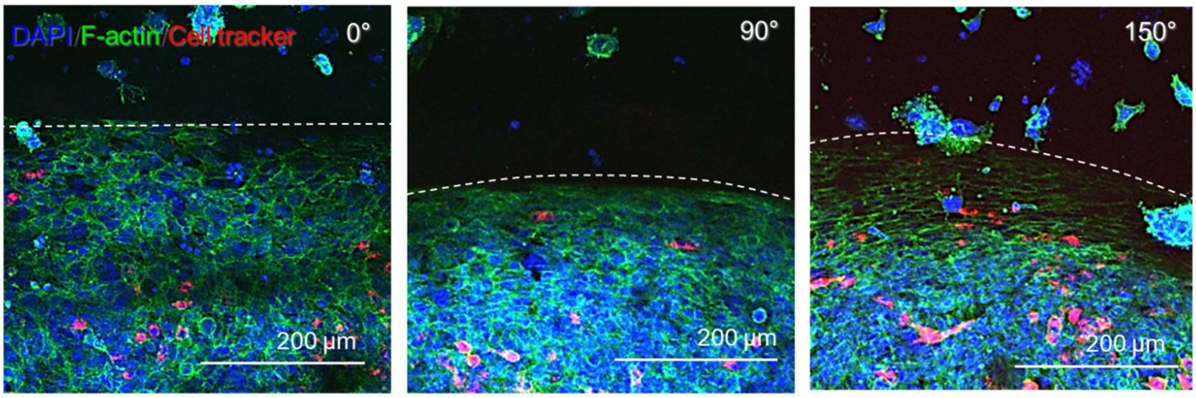

Supplementary Figure 15. Tumor cell adhesion on the confluent cerebrovascular wall. The tumor cells preferentially adhere to the endothelial wall at the curved portion. The scale bars are 500 μm (left) and 50 μm (right).

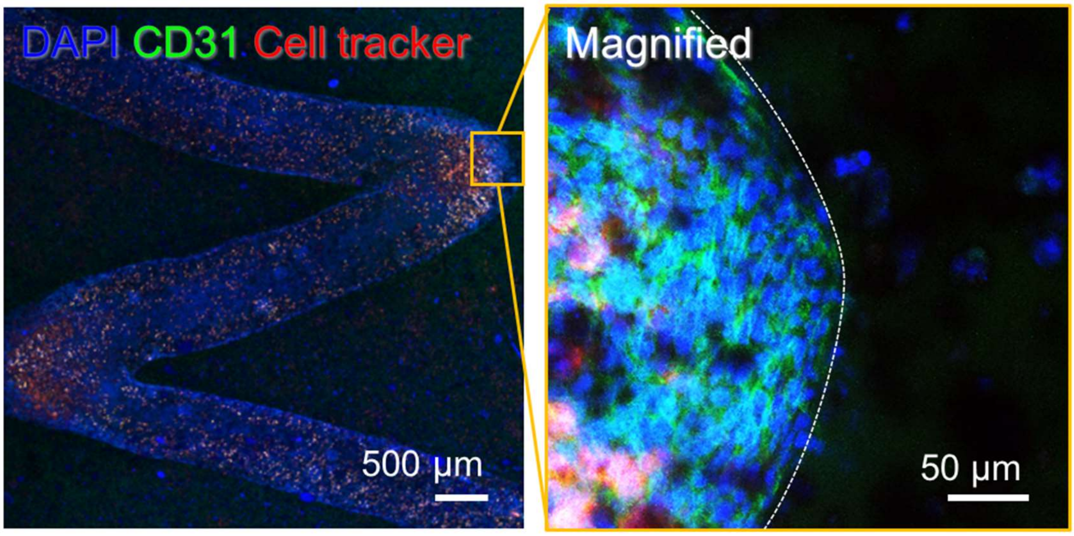

Supplementary Figure 16. Extravasation of introduced tumor cells in multilayered cerebrovascular conduits over time.

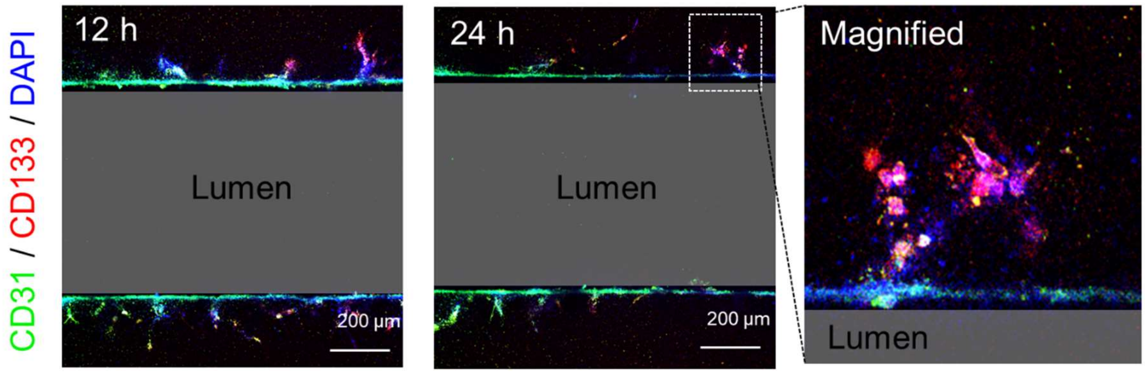

Supplementary Figure 17. Tumor cell extravasation from a multilayered cerebrovascular conduit (MCC). The tumor cells in metastatic stages including adhesion, extravasation, and colonization are observed in the MCC.

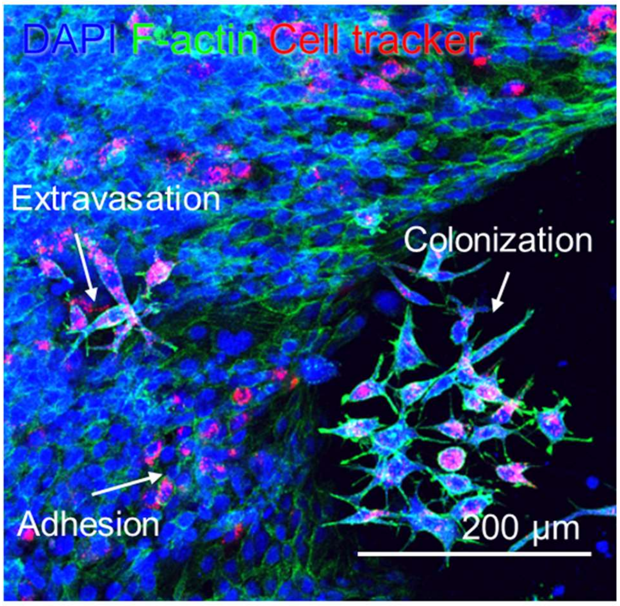

Supplementary Figure 18. Blocking cell adhesion molecules and cancer cell adhesion. a) Prior to introduction of cancer cells and the treatment of ICAM-1 function-blocking antibody ($C^- B^-$), the activated signal of the endothelial cells is not detected. After 24 h of treating cancer cells without the function-blocking antibody ($C^+ B^-$), some cancer cells adhere to the endothelium wall, and elongated their bodies. However, when the function of the adhesion molecules is blocked ($C^+ B^+$), a relatively small number of the adhered cancer cells with round-shaped bodies is observed. b) In the case of VCAM-1, blocking the adhesion molecules suppressed the tumor metastatic progression. c) The number of adhered circulating tumor cells is quantitatively measured. The results show mean ± SD from $n = 3$ samples. d) The aspect ratio of the adhered tumor cells is calculated. The results are compiled from $n = 30$ ($C^+ B^-$) and $n = 71$ ($C^+ B^+$) cells. The significance is determined using two tailed t-test (**, $p \leq 0.01$). Source data are provided as a Source Data file.

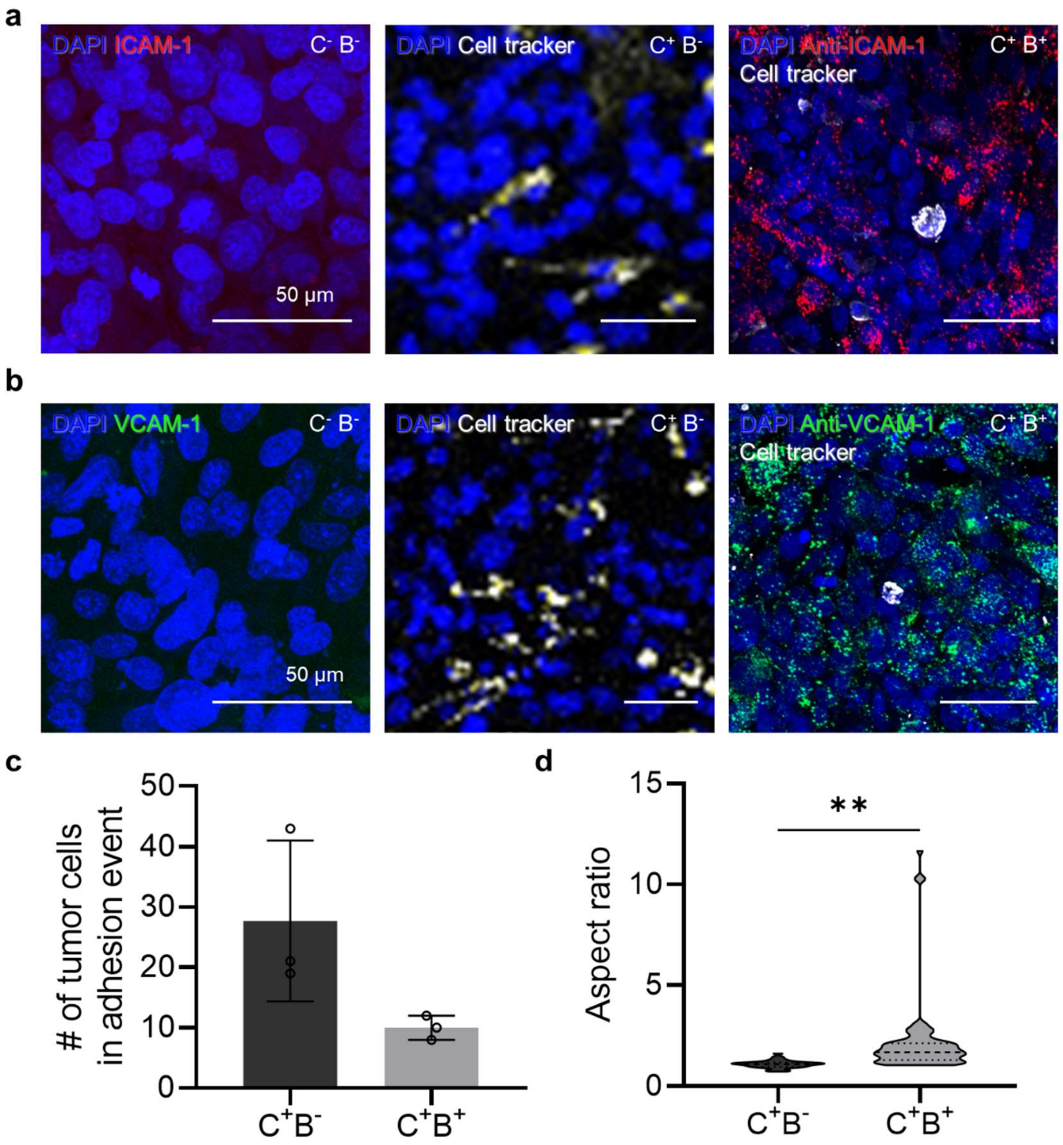

Supplementary Figure 19. Expression of adhesion proteins in multilayered cerebrovascular conduits in response to the different types of cancer cells. The results show mean ± SD from $n = 3$ samples. The significance is determined using two tailed t-test (*, $p \leq 0.05$). Source data are provided as a Source Data file.

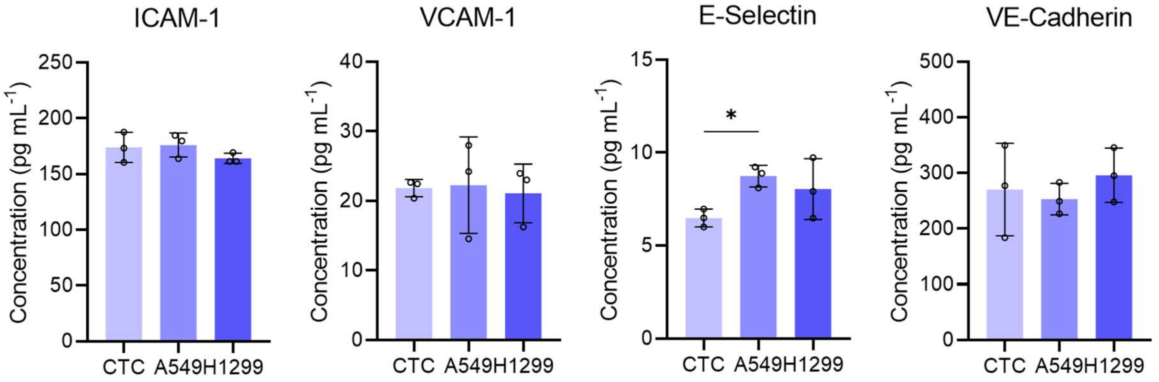

Supplementary Figure 20. Fluid dynamics simulation of pressure in the metastatic cancer model.

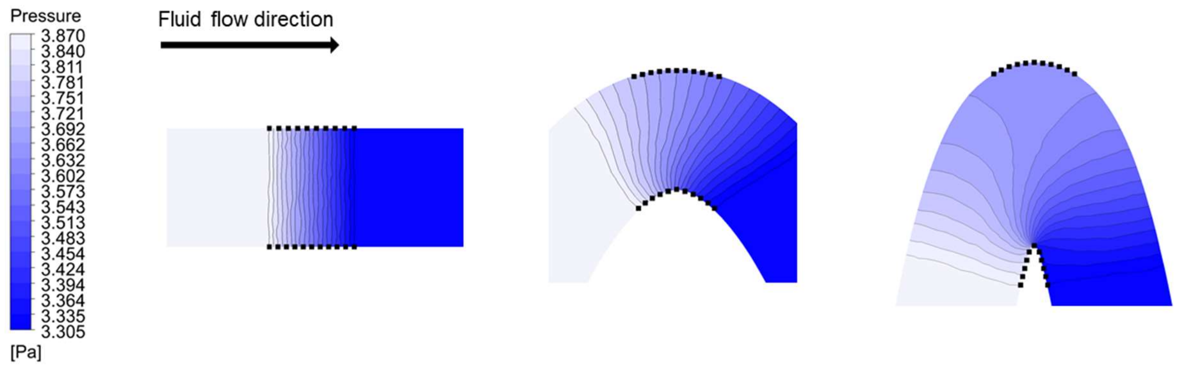

Supplementary Figure 21. Generation of the pressure gradient at different fluid flow velocities. The radial pressure gradient is induced when the input fluid velocity exceeds ~20 mm s^{-1} in bent tubes.

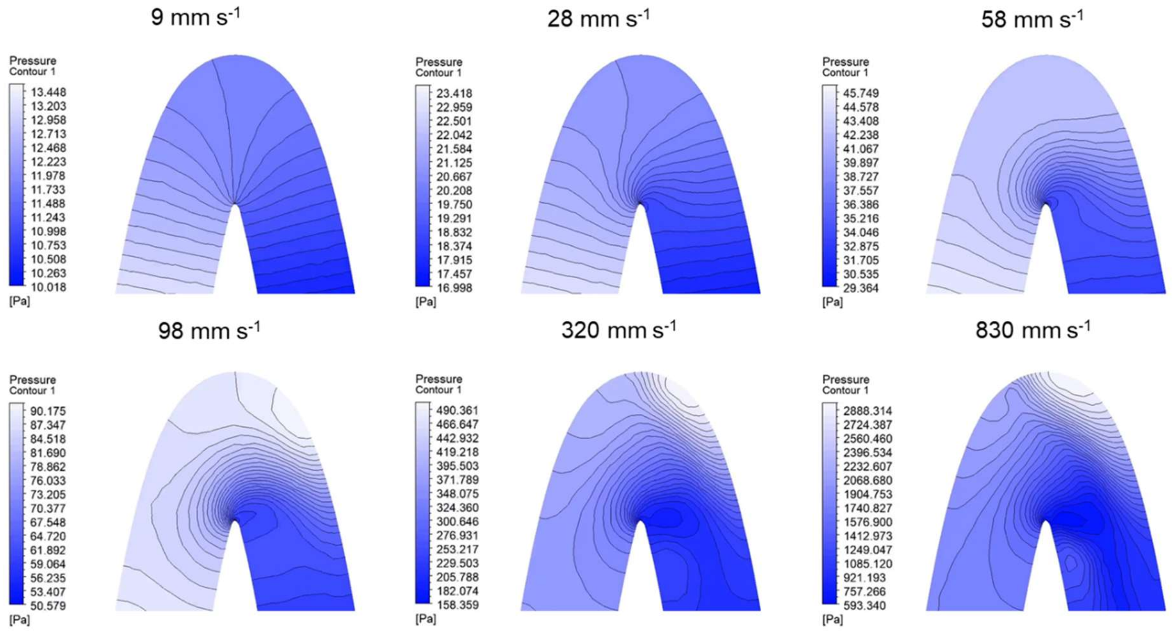

Supplementary Figure 22. Quantitative reverse-transcription PCR results demonstrating the effects of coculture and vascular curvatures on metastatic brain cancer development. A549 cells are circulated in four experimental groups: the straight multilayered cerebrovascular conduits (MCCs) with endothelial cell monoculture (SE), the straight MCCs with endothelial cell–stromal cell coculture (SES), the curved MCCs with endothelial cell monoculture (CE), and the curved MCCs with endothelial cell–stromal cell coculture (CES). The expression of key markers associated with B) endothelial barrier function (*ICAM-1*, *ZO-1*, and *Occludin*), C) epithelial–mesenchymal transition (*MMP-2*, *MMP-9*, and *TWIST*), D) neuroinflammatory responses (*TNF*, *IL-6*, *IL-8*, and *GFAP*), and E) tumorigenesis (*HGF*, *CX-43*) are analyzed. The results show mean ± SD from $n = 3$ samples. The significance is determined using two tailed t-test and ordinary one-way ANOVA (***, $p \leq 0.001$; **, $p \leq 0.01$; *, $p \leq 0.05$; ns, no significance). Source data are provided as a Source Data file.

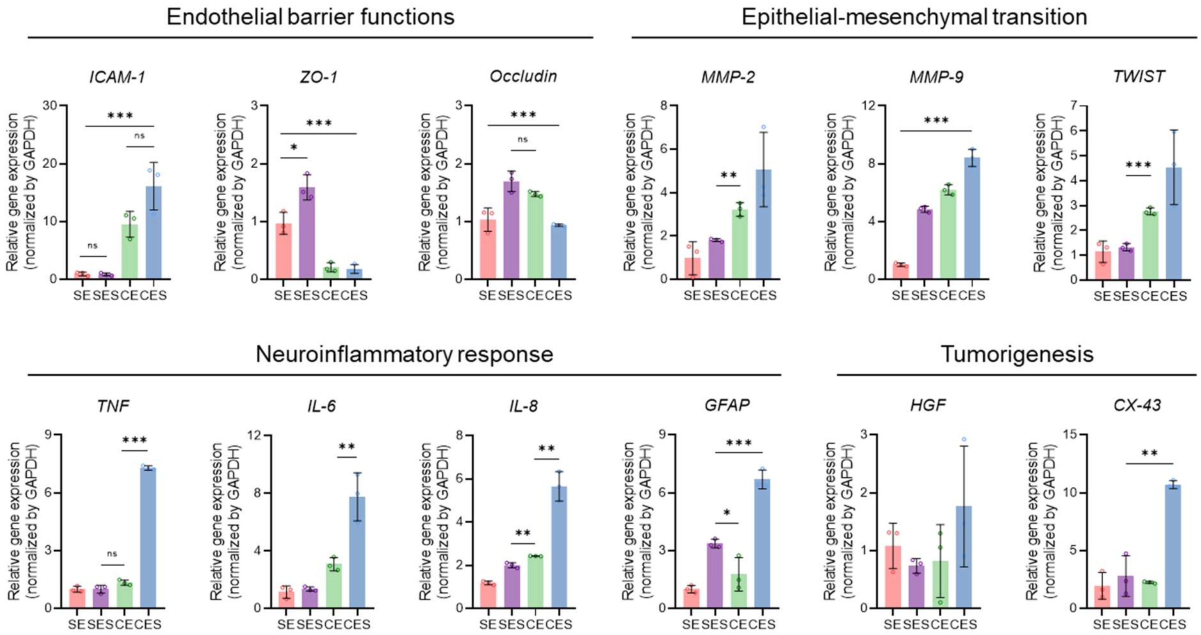

Supplementary Figure 23. The bioreactor set-up for development of an *in vitro* metastatic cancer model. a) A rocker shaker is used for the stabilization of the 3D bioprinted multilayered cerebrovascular conduits (MCCs) model. b) A peristaltic pump is connected for the fully mature brain tissue formation and the generation of the hemodynamic force for the study of the metastatic cancer progression.

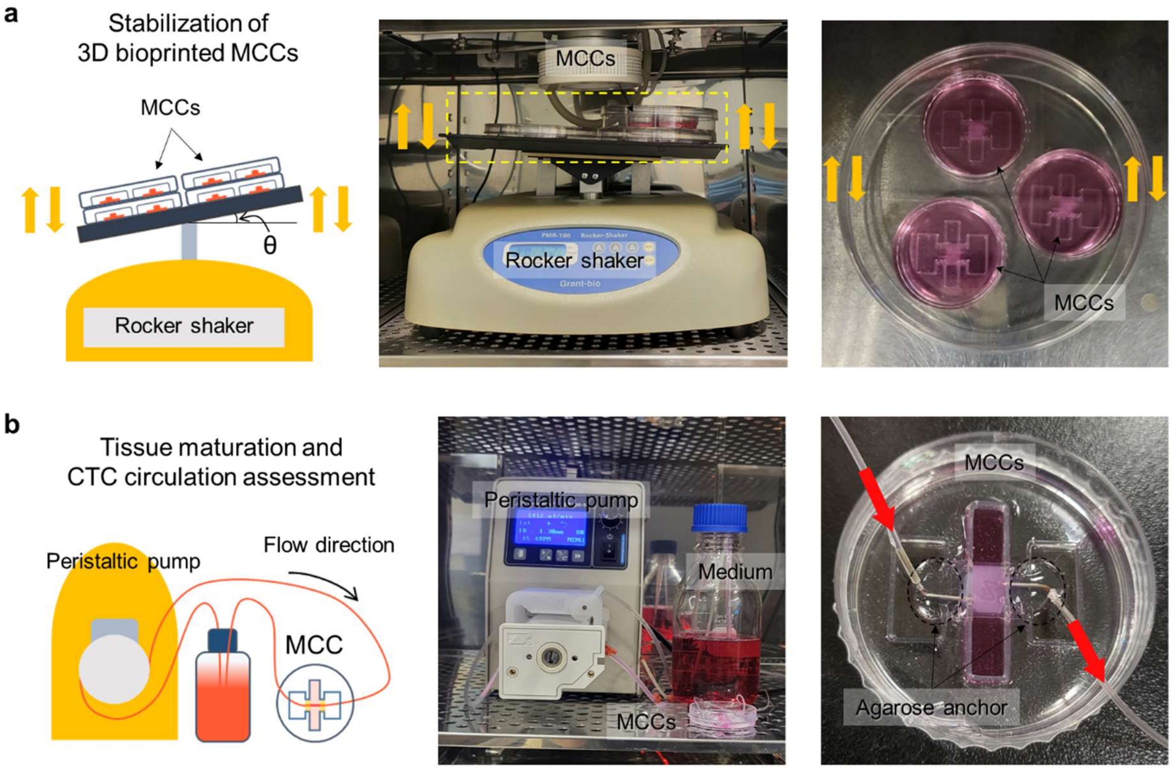

Supplementary Table 1. Protein expression level of cell adhesion molecules. The results are compiled from *n* = 4 samples. Source data are provided as a Source Data file.

Target protein ($pg\ mL^{-1}$)		E-selectin	VCAM-1	ICAM-1	VE-Cadherin
CTC^{-}	0°	3.54 ± 0.31	11.41 ± 2.13	76.55 ± 10.38	553.56 ± 12.09
	90°	3.39 ± 0.56	10.64 ± 3.09	85.16 ± 3.18	540.36 ± 168.89
	150°	3.273 ± 0.23	11.96 ± 1.74	82.81 ± 6.68	574.86 ± 109.44
CTC^{+}	0°	3.33 ± 0.34	14.73 ± 0.62	79.79 ± 5.87	489.17 ± 54.72
	90°	5.42 ± 0.50	17.00 ± 1.81	144.21 ± 12.31	368.24 ± 11.97
	150°	6.71 ± 0.59	21.83 ± 1.25	174.03 ± 19.14	266.86 ± 117.13

Supplementary Table 2. Cellular information of circulating tumor cells.

Cell type	Source	Donor
Human Lung Circulating Tumor cells	Human lung peripheral blood	Caucasia, Female, 62-year-old, metastatic lung cancer

Supplementary Table 3. Quantitative reverse-transcription PCR primer sequences.

Target gene	Forward primer sequence (5'→3')	Reverse primer sequence (5'→3')
GAPDH	GTCTCCTCTGACTTCAACAGCG	ACCACCCTGTTGCTGTAGCCAA
ZO-1	GTCCAGAATCTCGGAAAAGTGCC	CTTTCAGCGCACCATACCAACC
OCLN	ATGGCAAAGTGAATGACAAGCGG	CTGTAACGAGGCTGCCTGAAGT
VE-Cadherin	GAAGCCTCTGATTGGCACAGTG	TTTTGTGACTCGGAAGAACTGGC
CD31	AAGTGGAGTCCAGCCGCATATC	ATGGAGCAGGACAGGTTCAGTC
ICAM-1	AGCGGCTGACGTGTGCAGTAAT	TCTGAGACCTCTGGCTTCGTCA
MMP2	AGCGAGTGGATGCCGCCTTTAA	CATTCCAGGCATCTGCGATGAG
MMP9	GCCACTACTGTGCCTTTGAGTC	CCCTCAGAGAATCGCCAGTACT
TWIST	GCCAGGTACATCGACTTCCTCT	TCCATCCTCCAGACCGAGAAGG
TNF	CTCTTCTGCCTGCTGCACTTTG	ATGGGCTACAGGCTTGTCACTC
IL-6	AGACAGCCACTCACCTCTTCAG	TTCTGCCAGTGCCTCTTTGCTG
IL-8	GAGAGTGATTGAGAGTGGACCAC	CACAACCCTCTGCACCCAGTTT
GFAP	CTGGAGAGGAAGATTGAGTCGC	ACGTCAAGCTCCACATGGACCT
CX-43	GGAGATGAGCAGTCTGCCTTTC	TGAGCCAGGTACAAGAGTGTGG
HGF	GAGAGTTGGGTTCTTACTGCACG	CTCATCTCCTCTTCCGTGGACA
