# Supplementary material for: 3D bioprinted multilayered cerebrovascular conduits to study cancer extravasation mechanism related with vascular geometry
Source: Nat Commun. 2023 Nov 24;14:7696. doi: 10.1038/s41467-023-43586-4 (PMC10673893; doi:10.1038/s41467-023-43586-4)
Supplement: Supplementary file 3 — Reporting Summary [file 41467_2023_43586_MOESM3_ESM.pdf]

nature portfolio

Corresponding author(s): Prof. Byoung Soo Kim and Prof. Dong-Woo Cho

Last updated by author(s): Oct 23, 2023

Reporting Summary

Nature Portfolio wishes to improve the reproducibility of the work that we publish. This form provides structure for consistency and transparency in reporting. For further information on Nature Portfolio policies, see our Editorial Policies and the Editorial Policy Checklist.

Statistics

For all statistical analyses, confirm that the following items are present in the figure legend, table legend, main text, or Methods section.

n/a	Confirmed	
☐	☒	The exact sample size (*n*) for each experimental group/condition, given as a discrete number and unit of measurement
☐	☒	A statement on whether measurements were taken from distinct samples or whether the same sample was measured repeatedly
☐	☒	The statistical test(s) used AND whether they are one- or two-sided<br>*Only common tests should be described solely by name; describe more complex techniques in the Methods section.*
☒	☐	A description of all covariates tested
☒	☐	A description of any assumptions or corrections, such as tests of normality and adjustment for multiple comparisons
☐	☒	A full description of the statistical parameters including central tendency (e.g. means) or other basic estimates (e.g. regression coefficient) AND variation (e.g. standard deviation) or associated estimates of uncertainty (e.g. confidence intervals)
☐	☒	For null hypothesis testing, the test statistic (e.g. *F*, *t*, *r*) with confidence intervals, effect sizes, degrees of freedom and *P* value noted<br>*Give P values as exact values whenever suitable.*
☒	☐	For Bayesian analysis, information on the choice of priors and Markov chain Monte Carlo settings
☒	☐	For hierarchical and complex designs, identification of the appropriate level for tests and full reporting of outcomes
☒	☐	Estimates of effect sizes (e.g. Cohen's *d*, Pearson's *r*), indicating how they were calculated

Our web collection on statistics for biologists contains articles on many of the points above.

Software and code

Policy information about availability of computer code

Data collection	Rheology data was collected using TRIOS Software (3.1.0.3538). qRT-PCR data was collected using StepOne and StepOnePlus software. Fluorescent intensity was measured using ImageJ software (ImageJ 1.53k). Computational fluid dynamics simulation data was obtained using ANSYS software (2023 R1).
Data analysis	Data analysis was performed using GraphPad Prism (7.03).

For manuscripts utilizing custom algorithms or software that are central to the research but not yet described in published literature, software must be made available to editors and reviewers. We strongly encourage code deposition in a community repository (e.g. GitHub). See the Nature Portfolio guidelines for submitting code & software for further information.

Data

Policy information about availability of data

All manuscripts must include a data availability statement. This statement should provide the following information, where applicable:

- Accession codes, unique identifiers, or web links for publicly available datasets
- A description of any restrictions on data availability
- For clinical datasets or third party data, please ensure that the statement adheres to our policy

The mass spectrometry proteomics data have been deposited to the ProteomeXchange Consortium via the PRIDE partner repository with the dataset identifier PXD046191. Source data are provided with this paper.

Research involving human participants, their data, or biological material

Policy information about studies with human participants or human data. See also policy information about sex, gender (identity/presentation), and sexual orientation and race, ethnicity and racism.

Reporting on sex and gender	N/A
Reporting on race, ethnicity, or other socially relevant groupings	N/A
Population characteristics	N/A
Recruitment	N/A
Ethics oversight	N/A

Note that full information on the approval of the study protocol must also be provided in the manuscript.

Field-specific reporting

Please select the one below that is the best fit for your research. If you are not sure, read the appropriate sections before making your selection.

- [x] Life sciences
- [ ] Behavioural & social sciences
- [ ] Ecological, evolutionary & environmental sciences

For a reference copy of the document with all sections, see nature.com/documents/nr-reporting-summary-flat.pdf

Life sciences study design

All studies must disclose on these points even when the disclosure is negative.

Sample size	The sample sizes were equal or larger than 3 in all cases, which are similar to those generally employed in the field.
Data exclusions	No data have been excluded from the experiments unless apparent failures, such as cell contamination.
Replication	Experimental assays were performed at least three independent replicates, and all attempts at replication were successful.
Randomization	Randomization was not a relevant feature as we were applying a uniform set.
Blinding	Blinding was not a relevant feature as we were applying a uniform set.

Reporting for specific materials, systems and methods

We require information from authors about some types of materials, experimental systems and methods used in many studies. Here, indicate whether each material, system or method listed is relevant to your study. If you are not sure if a list item applies to your research, read the appropriate section before selecting a response.

Materials & experimental systems

n/a	Involved in the study	
[ ]	[x]	Antibodies
[ ]	[x]	Eukaryotic cell lines
[x]	[ ]	Palaeontology and archaeology
[x]	[ ]	Animals and other organisms
[x]	[ ]	Clinical data
[x]	[ ]	Dual use research of concern
[x]	[ ]	Plants

Methods

n/a	Involved in the study	
[x]	[ ]	ChIP-seq
[x]	[ ]	Flow cytometry
[x]	[ ]	MRI-based neuroimaging

Antibodies

Antibodies used	Anti-CD31 antibody (ab9498; 1:500), anti-Collagen IV antibody (ab6586; 1:200), and anti-MAP2 antibody (ab32454, 1:200) were purchased from Abcam. Anti-ICAM-1 antibody (BBA3; 20 ug/mL) and anti-VCAM-1 antibody (BBA5; 20 ug/mL) were purchased from R&D systems. Anti-MAP2 antibody (M4403; 1:500), anti-Laminin antibody (L9393; 1:200) were purchased from Sigma-Aldrich. Anti-GFAP antibody (MA5-12023; 1:200), anti-TuJ1 antibody (32-2600; 1:200), anti-ZO-1 antibody (33-9100; 1:200), and anti-rabbit (A11012, A11008; 5ug/mL) or anti-mouse (A11001, A11005; 5ug/mL) IgG fluorescent secondary antibodies were purchased from Invitrogen.

Validation	Anti-CD31 antibody (ab9498; Abcam): https://www.abcam.com/products/primary-antibodies/cd31-antibody-jc70a-ab9498.html<br><br>Anti-Collagen IV antibody (ab6586; Abcam): https://www.abcam.com/products/primary-antibodies/collagen-iv-antibody-ab6586.html<br><br>Anti-MAP2 antibody (ab32454, Abcam):https://www.abcam.com/products/primary-antibodies/map2-antibody-neuronal-marker-ab32454.html<br><br>Anti-ICAM-1 antibody (BBA3; R&D systems): https://www.rndsystems.com/products/human-icam-1-cd54-antibody-bbig-i1-11c81_bba3, https://www.rndsystems.com/products/antibodies<br><br>Anti-VCAM-1 antibody (BBA5; R&D systems):https://www.rndsystems.com/products/human-vcam-1-cd106-antibody-bbig-v1_bba5, https://www.rndsystems.com/products/antibodies<br><br>Anti-MAP2 antibody (M4403; Sigma-Aldrich): https://www.sigmaaldrich.com/KR/en/product/sigma/m4403<br><br>Anti-Laminin antibody (L9393; Sigma-Aldrich): https://www.sigmaaldrich.com/KR/en/product/sigma/l9393<br><br>Anti-GFAP antibody (MA5-12023; Invitrogen): https://www.thermofisher.com/antibody/product/GFAP-Antibody-clone-ASTRO6-Monoclonal/MA5-12023<br><br>anti-TuJ1 antibody (32-2600; Invitrogen):https://www.thermofisher.com/antibody/product/beta-Tubulin-Antibody-clone-2-28-33-Monoclonal/32-2600:<br><br>anti-ZO-1 antibody (33-9100; nvitrogen): https://www.thermofisher.com/antibody/product/ZO-1-Antibody-clone-ZO1-1A12-Monoclonal/33-9100<br><br>anti-rabbit IgG fluorescent secondary antibody (A11012; Invitrogen): https://www.thermofisher.com/antibody/product/Goat-anti-Rabbit-IgG-H-L-Cross-Adsorbed-Secondary-Antibody-Polyclonal/A-11012<br><br>anti-rabbit IgG fluorescent secondary antibody (A11008; Invitrogen): https://www.thermofisher.com/antibody/product/Goat-anti-Rabbit-IgG-H-L-Cross-Adsorbed-Secondary-Antibody-Polyclonal/A-11008<br><br>anti-mouse IgG fluorescent secondary antibody (A11001; Invitrogen): https://www.thermofisher.com/antibody/product/Goat-anti-Mouse-IgG-H-L-Cross-Adsorbed-Secondary-Antibody-Polyclonal/A-11001<br><br>anti-mouse IgG fluorescent secondary antibody (A11005; Invitrogen): https://www.thermofisher.com/antibody/product/Goat-anti-Mouse-IgG-H-L-Cross-Adsorbed-Secondary-Antibody-Polyclonal/A-11005

Eukaryotic cell lines

Policy information about cell lines and Sex and Gender in Research

Cell line source(s)	Human Brain Microvascular Endothelial Cells (Innoprot, P10361) derived from human brain, Human Brain Vascular Pericytes (SciencCell, 1200) isolated from human brain, ReNcell CX Human Neural Progenitor Cell Line (Sigma-Aldrich, SCC007), and Human lung Circulating Tumor Cell (Celprogen, 36107-34CTC), H1299 (Korean Cell Line Bank), A549 form human lung (male, 59 years old, Caucisian; Korean Cell Line Bank) were used.
Authentication	None of cell lines were authenticated.
Mycoplasma contamination	The cell lines were not tested for mycoplasma contamination.
Commonly misidentified lines<br>(See ICLAC register)	No commonly misidentified cell lines were used in this study.
